# Optimization of Baker’s Yeast Production on Date Extract Using Response Surface Methodology (RSM)

**DOI:** 10.3390/foods6080064

**Published:** 2017-08-07

**Authors:** Mounira Kara Ali, Nawel Outili, Asma Ait Kaki, Radia Cherfia, Sara Benhassine, Akila Benaissa, Noreddine Kacem Chaouche

**Affiliations:** 1 Laboratoire de Mycologie, de Biotechnologie et de l’Activité Microbienne (LaMyBAM), Département de Biologie Appliquée, Faculté des Sciences de la Nature et de la Vie, Université Frères Mentouri Constantine 1, Constantine 25000, Algeria; cherfiarr@yahoo.fr (R.C.); sarabenhassine@gmail.com (S.B.); nkacemchaouche@yahoo.fr (N.K.C.); 2 Laboratoire de Génie des Procédés de l’Environnement, Faculté du Génie des Procédés, Université Constantine 3, Constantine 25000, Algeria; out.nawel@gmail.com (N.O.); akilabenaissa@yahoo.fr (A.B.); 3 Département de Biologie, Faculté des Sciences de la Nature et de la Vie, Université M’hamed Bougera, Boumerdess 35000, Algeria; askaki.biotechno@gmail.com

**Keywords:** *Saccharomyces cerevisiae*, biomass, date extract, optimization, response surface methodology, kinetic models

## Abstract

This work aims to study the production of the biomass of *S. cerevisiae* on an optimized medium using date extract as the only carbon source in order to obtain a good yield of the biomass. The biomass production was carried out according to the central composite experimental design (CCD) as a response surface methodology using Minitab 16 software. Indeed, under optimal biomass production conditions, temperature (32.9 °C), pH (5.35) and the total reducing sugar extracted from dates (70.93 g/L), *S. cerevisiae* produced 40 g/L of their biomass in an Erlenmeyer after only 16 h of fermentation. The kinetic performance of the *S. cerevisiae* strain was investigated with three unstructured models i.e., Monod, Verhulst, and Tessier. The conformity of the experimental data fitted showed a good consistency with Monod and Tessier models with *R^2^* = 0.945 and 0.979, respectively. An excellent adequacy was noted in the case of the Verhulst model (*R^2^* = 0.981). The values of kinetic parameters (*Ks*, *X_m_*, *μ_m_*, *p* and *q*) calculated by the Excel software, confirmed that Monod and Verhulst were suitable models, in contrast, the Tessier model was inappropriately fitted with the experimental data due to the illogical value of *Ks* (−9.434). The profiles prediction of the biomass production with the Verhulst model, and that of the substrate consumption using Leudeking Piret model over time, demonstrated a good agreement between the simulation models and the experimental data.

## 1. Introduction

For thousands of years, micro-organisms have been spontaneously used in human food preparation. However, scientists did not initiate studying these living beings until the appearance of the microscope in 1680. Among microorganisms widely studied and used in diverse biotechnological applications, the yeast *S. cerevisiae* was mentioned [1,2,3]. This yeast species was known formerly for its particular exploitation in the production of wine, beer and bread. Recently, it has been used as a “cell factory”, able to synthesize a large spectrum of bioactive molecules as recombinant proteins, antibiotics and bioethanol [4,5,6,7]. Algeria records a remarkable lack in beet and cane molasses production, indeed, it imports about 18,000 and 13,000 tons per year of each one, respectively, and also imports the yeast strain *S. cervisiae* used as a baker’s leaven [8]. However, Algeria has an enormous potential of dates [9,10]. In addition, the production of *S. cerevisiae* biomass from a low quality date variety could constitute an economic carbon source, especially considering that the production of dates is a bountiful in Algeria. The main objective of the present work is to study the optimization of *S. cerevisiae* biomass production, using date extract as a sole carbon source.

The traditional technique used for optimizing a multivariable fermentation process is difficult and does not take the alternative effects between components into consideration [11,12]. Recently, many statistical experimental design methods have been employed in bioprocess optimization [13,14,15,16]. Among these, the central composite experimental design (CCD) is the most suitable for identifying the individual variables to optimize a multivariable system [17,18]. This method was used to optimize many fermentation process, such as acids, antibiotics, enzymes, biomass and ethanol production by several micro-organisms types [19,20,21,22]. Furthermore, it was used in design, analysis, and in unit operations. The advantages of this method are the reduction of the number of experiments, reagents, time, financial input and energy [23]. The present work was conducted following these steps: (a) selecting the optimum conditions of three parameters (temperature; initial pH, and sugar concentration extracted from dates) to obtain a high yield of *S. cerevisiae* cells growth using a surface response methodology; (b) exploit the date extract as the sole carbon source for the production of *S. cerevisiae* at optimized conditions; (c) predict the biomass production process by unstructured kinetic models.

## 2. Materials and Methods

### 2.1. Origin and Reactivation of theYeast S. cerevisiae

The yeast used in this study has a commercial origin in fact, it is produced by the factory Lesaffre. The lyophilized form of *S. cerevisiae* was chosen due to several advantages, such as its availability, rapid growth, resistance to contaminants, easy cultivation, ability to consume most sugar and high yield production. The yeast was reactivated on agar plates containing YPGA medium composed of yeast extract 10 g/L, peptone 10 g/L, glucose 20 g/L, agar 20 g/L with a pH 6, incubated at 30 °C for 24 h. After development, the yeast was analyzed by macroscopic and microscopic characteristics in order to confirm its aspect.

### 2.2. Preparation of Dates Extract

**Figure 1:**
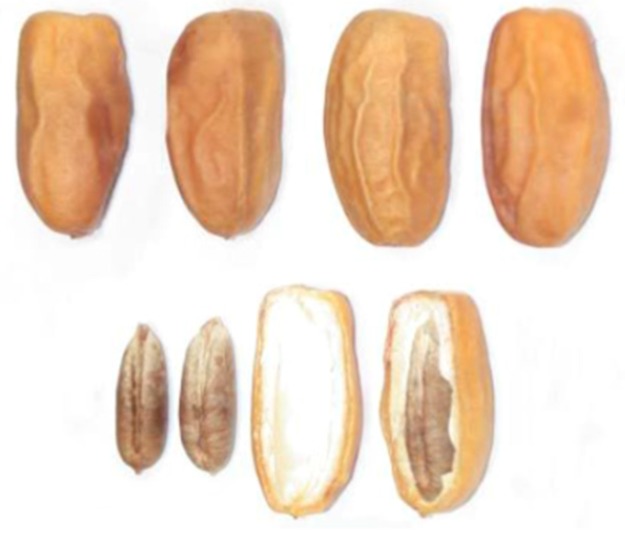
Dates *Mech-Degla.*

The date variety used in this study is very widespread in the south-eastern region of Algeria. It is the dry variety *Mech-Degla*. This variety has a sub-cylindrical shape, slightly narrowed at its tip. It is lightly beige-tinted. The epicarp is wrinkled, shiny and brittle. The mesocarp is not very fleshy of a dry consistency and fibrous texture (Figure 1) [8]. The choice of this variety is justified by its abundance at the national level, low market value, ease of preservation (dry date) and richness in sugars. This variety is therefore a favorable substrate for the yeast growth and development.

To prepare the date extract, 1 kg of this date is washed, peeled and placed in 5 L of distilled water and then boiled at 100 °C for one hour in order to extract the sugars. The obtained extract is filtered through muslin to remove the large particles, and then the solution is centrifuged at 5000 rpm for 5 min. The supernatant obtained constitutes the date extract [24].

### 2.3. Preparation of Culture Medium Based on the Dates Extract and Inoculums

The method cited by Kocher and Uppal [25] was used with minor modifications. The obtained date extract from the above preparation was supplemented by mineral salts: magnesium sulfate 0.44 g, urea 12.70 g, and ammonium sulfate 5.30 g. Finally, the medium was distributed in an Erlenmeyer of 250 mL with a ratio of 100 mL per flask and sterilized at 120 °C for 20 min. The pre-culture was obtained by inoculating two colonies of the yeast *S. cerevisiae* in 250 mL shake flasks containing 100 mL of dates extract, mentioned above. The pre-culture was incubated at 30 °C for 3 h, and used further as inoculums for the yeast biomass production.

### 2.4. Statistical Design of Experiments

#### 2.4.1. Factor Selection and Organization of Experiments

The organization of the experiments was carried out using the experimental design obtained by the central composite experimental design (CCD). Three independent variables were selected (temperature, initial pH and concentration of sugars extracted from dates). Table 1 shows the domain of study with coded levels and real values of studied variables.

The CCD matrix employed for three independent variables is given in Table 2. Each column represents the different variables (factors) and each line represents the different experiments (20).

#### 2.4.2. Effect Estimation

The real values *X* have been calculated according to Equation (1).

(1) $$X\;=\;\frac{x-x_{0}}{\Delta x}$$

Where *X*, is the coded value for the independent variable, *x*, is the natural value, *x_0_*, is the natural value at the center point and Δ*x*, is the step change value (the half of the interval (−1 +1)). The mathematical model describing the relation between dependent and independent variables for this process has the quadratic form for the experimental design used:

*Y*_i_ = *β*_0_ + β_1_*X*_1_ + *β*_2_*X*_2_ + *β*_3_*X*_3_ + *β*_11_*X*_1_² + *β*_22_*X*_2_² + *β*_33_*X*_3_² + *β*_12_*X*_1_*X*_2_ + *β*_13_*X*_1_*X*_3_ + *β*_23_*X*_2_*X*_3_ (2)

where *Y*_i_, is the predicted response (in our case, the Biomass production (g/L); *β*_0_, is offset term; *β*_1_, *β*_2_, *β*_3_ are the linear effects (showing the predicted response); *β*_11_, *β*_22_, *β*_33_ are the squared effects *β*_12_, *β*_13_, *β*_23_ are the interaction terms and *X*_1_, *X*_2_, *X*_3_ are the independent variables. The calculation of the effect of each variable and the establishment of a correlation between the response *Y*_i_ and the variables *X*, were performed using a Minitab 16 software (Minitab, Inc., State College, PA, USA).

#### 2.4.3. Statistical Analysis

The statistical analysis was performed using analysis of variance (ANOVA), in order to validate the square model regression. It included the following parameters: coefficient of determination *R**^2^*** Student test (*t*); Fisher test (*F*); and *p*-value. In our study, the statistical significance test level was set at 5% (probability (*p*) < 0.05).

### 2.5.Validation of Biomass Production in Optimum Medium

In order to confirm the optimized conditions obtained by the central composite design, an experiment was carried out on 250 mL shake flasks. To do this, 100 mL of date extract (mentioned above) was seeded with 11 mL of the yeast pre-culture and the pH of the medium was adjusted to 5.35 (optimum value). Shake flasks were sterilized at 120 °C for 20 min, and incubated at 32.9 °C (optimum value) for 18 h.

### 2.6. Analytical Techniques

#### 2.6.1. Determination of Total Reducing Sugars

The total reducing sugars were determined according to the method of Dubois et al. [26], with minor modifications. The sample was filtered, and 1 mL of it was transferred to a glass tube. Then 0.6 mL of 5% (*w/v*) phenol and 3.6 mL of 98% sulfuric acid were added. The mixture was shaken and incubated at room temperature for 30 min; sugar gives a cream yellow whose intensity is proportional to the amount of total sugars. The absorbance was measured at 490 nm. A calibration curve was established using glucose as the standard.

#### 2.6.2. Determination of Biomass Concentration

The measurement of biomass was followed by estimation of cell dry weight, expressed in g/L. one mL of yeast culture was centrifuged at 5000 rpm for 5 min. The supernatant obtained was washed twice with water and dried by incubation at 105 °C until at a constant weight [27].

### 2.7. Modeling

Unstructured kinetic models using Monod, Verhulst, and Tessier [28] (Table 4) have been implemented to fit the experimental data. Kinetic parameters (*µ_max_*, *Ks* and *X_m_*), were determined using the curve fitting method of each model. The fitness evaluation of experimental data on cell growth by models was performed using Excel software (Microsoft, Redmond, WA, USA).

#### Profile Prediction of Biomass and Substrate Concentration

To predict the experimental profile of biomass of *S. cerevisiae* during time fermentation, the integration of the Verhulst model was used to give a sigmoidal variation of *X* as a function of *t*, which may represent both an exponential and a stationary phase (Equation (3)):

(3) $$X=\frac{X_{0}e^{\mu_{m}t}}{\left\{1-\left(X_{0}/\;X_{m}\right)\left(1-e^{\mu_{m}t}\right)\right\}}$$

In addition, the substrate model (Leudeking Piret) as described below (Equation (4)) was also applied to predict an experimental profile for total reducing sugars consumption by *S. cerevisiae* during time.

(4) $$-\frac{dS}{dt}=p\frac{dX}{dt}+qX$$

where *p* = 1/*Y_X/S_* and *q* is a maintenance coefficient.

Equation (4) is rearranged as follows:

(5) −dS=p dX+q∫X(t)dt

Substituting Equation (3) in Equation (5) and integrating with initial conditions (*S* = S_0_; *t* = 0) give the following Equation:

(6) $$S=S_{0}-pX_{0}\left\{\frac{e^{\mu_{m}t}}{\left\{1-\left(\frac{X_{0}}{X_{m}}\right)\left(1-e^{\mu_{m}t}\right)\right\}}-1\right\}-q\frac{X_{m}}{\mu_{m}}ln\{1-\frac{X_{0}}{X_{m}}\left(1-e^{\mu_{m}t})\right\}$$

## 3. Results and Discussion

Microbial growth is influenced by the culture medium constituents and the physico-chemical factors in particular, temperature, pH, and substrate concentration. Indeed, in the present study, the temperature, the initial pH and the concentration of the carbon source (total sugars extracted from dates) were supposed to optimize the biomass production of *S. cerevisiae* using the central composite experimental design. The biomass concentration over 16 h of fermentation varied with the change in temperature, initial pH and sugar concentration (Table 5).

Using the results obtained in diverse experiments, the correlation gives the influence of temperature (*X_1_*), initial pH (*X_2_*) and total sugar concentration (*X_3_*) on the response. This correlation is obtained by Minitab 16 software and expressed by the following second order polynomial (Equation (7)):

*Y_i_* = 40.074 −0.568*X_1_*− 0.090*X_2_* + 1.373*X_3_* − 5.999*X_1_*² − 4.248*X_2_*² − 5.893*X_3_*² − 0.070*X_1_X_2_* + 2.772*X_1_X_3_* − 1.925*X_2_X_3_* (7)

Table 6 shows the coefficient regression corresponding with *t* and *p*-values for all the linear, quadratic and interaction effects of parameters tested. A positive sign in the *t*-value indicates a synergistic effect, while a negative sign represents an antagonistic effect of the parameters on the biomass concentration [29].

**Figure 2:**
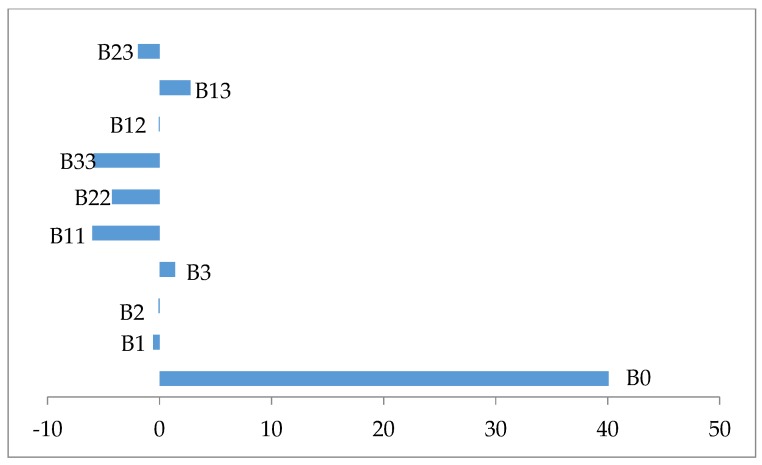
Variable effect signification on a biomass production.

The examination of Table 6 shows that all coefficient regression of the quadratic terms are statistically significant *p* ≤ 0.05 and negatively affect the biomass production (Figure 2). In contrast all coefficient regression of linear and interaction terms were statistically not significant *p* > 0.005, except the interaction term *X_1_X_3_*, which is significant *p* = 0.044 and has a synergistic effect on the response (Figure 2).

The analysis of variance (ANOVA) of the coefficient regression for the cell growth production (Table 7) demonstrates that the model is significant due to the *F*-value of 11.43 and the low probability *p* value (*p* = 0.000). Generally, the *F*-value with a low probability *p*-value indicates a high significance of the regression model [30].

**Figure 3:**
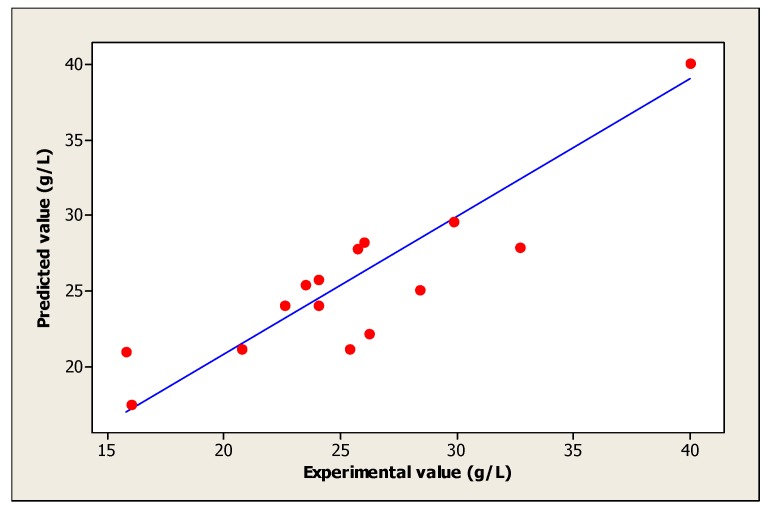
The fit between the model and experimental data of cell growth.

Moreover, the coefficient of determination (*R*^2^) measures the fit between the model and experimental data. Figure 3 was also determined to evaluate the regression model. In this study, the obtained value of *R*^2^ is 0.911 approximate to 1, which justifies an excellent consistency of the model [31]. On the other hand, the obtained *R*^2^ implies that 91.1% of the sample variation in the cell growth is attributed to the independent variables. This value indicates also that only 8.86% of the variation is not explained by the model.

According to the literature, the study proposed by Boudjemaet al. [22] was carried out using a design of experiment to describe the batch fermentation of bioethanol and biomass production on sweet cheese whey by *Saccharomyces cerevisiae* DIV13-Z087C0VS. The results showed a good agreement with experimental data (a low probability *p* value ≤ 0.000 and a good correlation coefficient (*R*^2^ = 0.914%), which confirms a high significance of the regression model. In addition, the study carried out by Bennamoun et al. [32] showed that the optimization of the medium components, which enhance the polygalacturonase activity of the strain *Aureobasidium pullulans*, was achieved with the aid of the same method used in the present study (response surface methodology). The obtained results showed a significance of the method used in comparison with the experimental data; a very low *p* value (0.001) and a high coefficient of determination (*R*^2^ = 0.9421).

**Figure 4:**
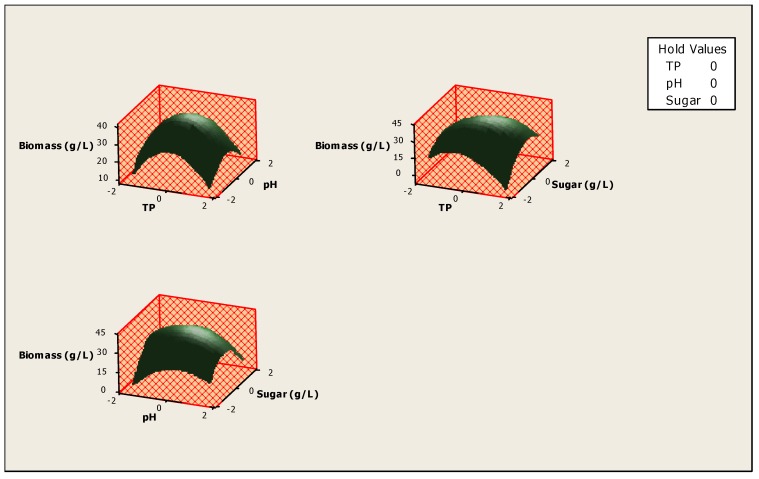
Surface plot for the effect of different parameters on biomass production.

**Figure 5:**
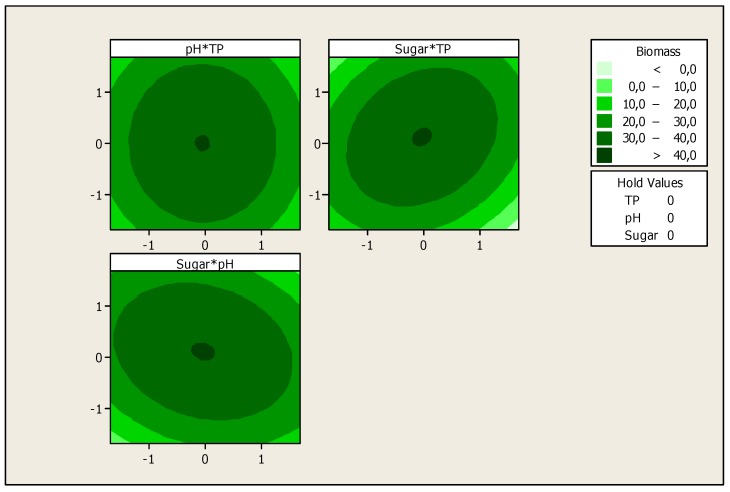
Isoresponse contour plot for the effect of the studied variables on biomass production.

The optimization of the response *Y_i_* (Biomass production) and the prediction of the optimum levels of temperature, initial pH and sugars concentration of fermentation were obtained. This optimization resulted in surface plots (Figure 4) and an isoresponse contour plot (Figure 5).

**Figure 6:**
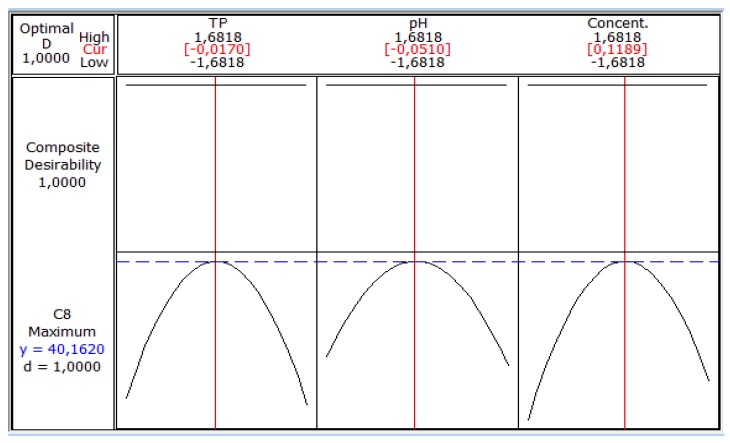
Coded values of optimal conditions on biomass production.

These figures show that there is an optimum, located at the center of the field of study. In addition, the use of the minitab optimizer will give exact values of the optimum operating conditions of the process (Figure 6).

Figure 6 shows the maximum biomass production by *S. cerevisiae* (40.162 g/L) corresponding to coded values of temperature (−0.0170), pH (−0.0510) and sugar concentration (0.1189).These values are equivalent to real values of 32.9 °C, 5.35 and 70.93 g/L, respectively. Jiménez Islas et al. [27] obtained the highest cell concentration of *S. cerevisiae* ATCC 9763 (7.9 g/L) after 26 h when the strain grew at 30 °C and pH 5.5.

**Figure 7:**
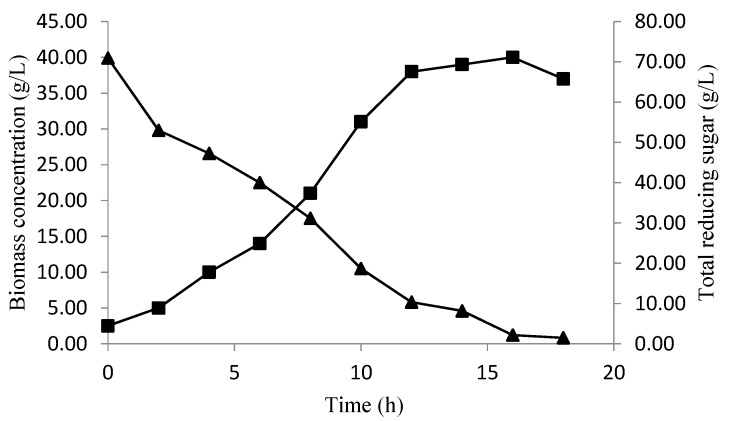
The biomass production (■), and total reducing sugar consumption (▲) over time at optimized conditions.

The validation of the baker’s yeast biomass concentration and total reducing sugar consumption, over time, at optimized conditions, are presented in Figure 7. In the beginning, the biomass concentration increased with a decrease in the sugar level, reaching the maximum (40 g/L) at 16 h of fermentation, which confirms the biomass obtained by the CCD predictions (40.1620) (Figure 6). After this period, the diminution of a biomass concentration was observed, which could be explained by the sugar consumption, which ran out after18 h of fermentation.

The same results were obtained by Nancib et al. [33], where the production of biomass from baker’s yeast *S. cerevisiae* on a medium containing date byproducts was 40 g/L. Khan et al. [34] used six different strains of *S*. *cerevisiae* in fermentation medium containing date extract (with 60% sugars), in addition to 2 g/L ammonium sulfate and 50 mg/L biotin. Their results showed that the theoretical yields were about 42.8%. In addition, Al Obaidi et al. [35] studied two substrates i.e, date syrup and molasses for the propagation of baker’s yeast strain *S. cerevisiae* on a pilot plant scale. The results showed that higher productivity of baker's yeast was observed when date extract was used. Other results were obtained in several studies using an alternative substrate of fermentation. In fact, the optimal biomass production (6.3 g/L) was depicted at 24 h using *Saccharomyces cerevisiae* DIV13-Z087C0VS on a medium containing sweet cheese as a sole carbon source [22]. On the other hand, the production of baker’s yeast from apple pomace gives a yield of 0.48 g/g [36]. Therefore, it was concluded from these studies that the medium containing the date extract as a sole carbon source is an excellent fermentation medium for baker’s yeast production.

The results of the kinetic parameters of *S. cerevisiae* growth with the different kinetic models based on the curve fitting method are presented in Table 8.

**Figure 8:**
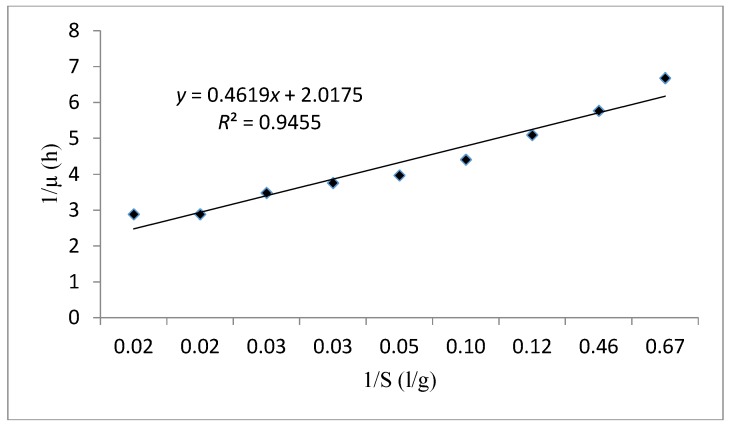
The Lineweaver Burk linear plot fitting the experimental data using the Monod kinetic model.

The curve fitting of cell growth using the Monod model (1/μ versus 1/S) is presented in Figure 8. Based on the results obtained in Table 8 for this model the *μ_max_* and *Ks* were evaluated as 0.496 h^-1^ and 0.228 g/L, respectively. These values indicate a rapid cell growth due to the high value of the specific growth rate and an elevated affinity between substrate consumption and cell growth thanks to the small half-saturation constant. In this case, *R*^2^ was also fitted on 0.945. According to the results obtained, the Monod kinetic model is an appropriate model to make the kinetic performance of this strain.

**Figure 9:**
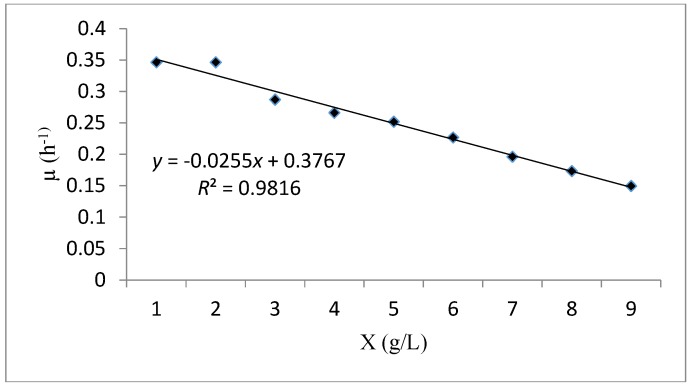
A plot fitting the experimental data using the Verhulst kinetic model.

Figure 9 illustrates the linear curve fitting (μ versus X) to examine the reliability of cell kinetic performance via the Verhulst model. The analysis of the results obtained showed that the experimental data of the cell growth and substrate consumption in batch system have an excellent fitness with this model (*R*^2^ = 0.981). The maximum specific growth rate (*μ_max_*) and the maximum concentration of biomass (*X_m_*), were 0.376 h^−1^ and 15.04 g/L respectively (Table 8). Higher values of these parameters indicated a rapid growth of the biomass which confirms the goodness of fit of the Verhulst model.

**Figure 10:**
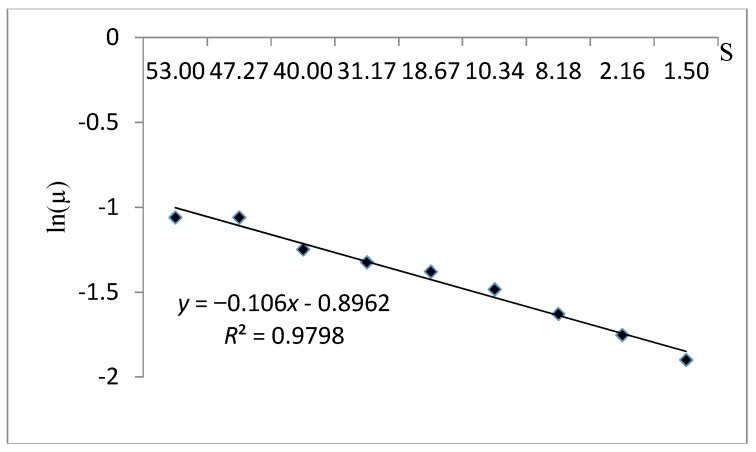
A plot fitting the experimental data using the Tessier kinetic model.

The kinetic behavior fitness of *S. cerevisiae* with the Tessier kinetic model is illustrated in Figure 10. The coefficient of correlation *R*^2^ equal to 0.979 and the estimation parameters (µ_max_ and Ks) shown in Table 8 were 0.408 and −9.434 respectively. The examination of the cell growth fitting curve with the Tessier kinetic model showed that, even though they were appropriate *R*^2^ and µ_max_ values, the model is not suitable with the experimental data due to the illogical value of the half-saturation constant (negative Ks).

The comparison between the three kinetic models tested in this study showed that the Verhulst kinetic model with *R*^2^ = 0.981 was the best and most appropriate model to explain *S. cerevisiae* growth and substrate utilization. Approximate results were obtained by Ardestani and Shafiei [37], who proved that the Verhulst kinetic model with *R*^2^ equal to 0.97 was the most appropriate to describe the biomass growth rate of *S. cerevisiae.* In contrast, Ardestani and Kasebkar [38], applied an unstructured kinetic model of *Aspergillus niger* growth and substrate uptake in a submerged batch culture and have confirmed that Monod and Verhulst kinetic models were not in an acceptable range to fit a growth of *Aspergillus niger.*

**Figure 11:**
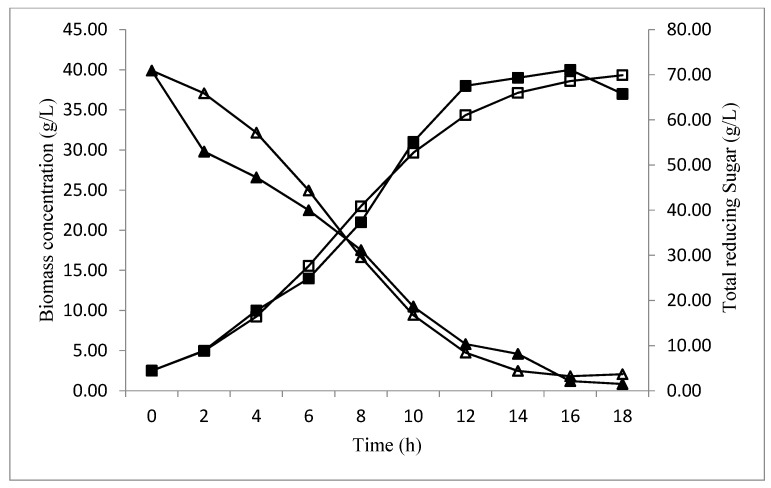
The comparison between predicted (□), experimental data (■) for biomass production of baker’s yeast; and predicted (∆), experimental data (▲), for total reducing sugar consumption.

A profile of biomass and total reducing sugar concentration during fermentation time is compared to the values predicted by the equations model obtained in Figure 11.

At the beginning of the fermentation, values of biomass between predicted and experimental data were approximately the same. However, after 10 h and until the end of the fermentation, the difference was remarkable. In fact, the values relative to biomass were inferior compared to the values predicted by the Verhulst model. The correlation coefficient is 0.992. As for total reducing sugar concentration, the values obtained by the Leudeking Piret model were lower than those predicted in the first 7 h only. After this period, total reducing sugar values were almost identical. The correlation coefficient is 0.984. In addition, the parameter values of *p* and *q* were optimized using the experimental data for substrate based on the square minimized between observed and predicted data. Excel software illustrated the values of *p* = 2.1235 and *q* = −0.0256 h^−1^. On the basis of these results, good correlation coefficients showed that the proposed Verhulst model and the Luedeking Piret model were adequate to explain the development of biomass production process on date extract. According to the literature, the study proposed by Kara Ali et al. [39] was carried out using the logistic empirical kinetic model and Leudeking Piret model to describe batch fermentation of *P. caribbica* on inulin. The results showed a good agreement with the experimental data (*R*^2^ = 0.91) for cell growth and (*R*^2^ = 0.95) for substrate consumption. In addition, the values of p and q were 14.735 and −0.077 1/h, respectively, thus, the model equations were found to represent an appropriate kinetic model for successfully describing yeast cell growth in batch fermentation. Another kinetic study proposed by Zajšek and Goršek [40] which used the unstructured models of batch kefir fermentation kinetics for ethanol production by mixed natural microflora confirmed that the growth of kefir grains could be expressed by a logistic function model, and it can be employed for the development and optimization of bio-based ethanol production processes. Furthermore, the study of Pazouki et al. [41] which illustrated the kinetic models of cell growth, substrate utilization and bio-decolorization of distillery waste water by *Aspergillus fumigatus* UB260. This study confirmed that the Logistic equation for the growth and the Leudeking Piret kinetic model for substrate utilization were able to fit the experimental data (*R*^2^ = 0.984). The coefficient equation were also calculated (*p* and *q*) their values were 1.41 (g/g) and 0.0007 (1/h) respectively.

## 4. Conclusions

Microbial fermentation is complex and it is quite difficult to understand its complete details process. The central composite design (CCD) proposed in this study seems pertinent to describe the optimum biomass production of *S. cerevisiae*. A second order polynomial model was developed to evaluate the quantitative effects of temperature, pH and reducing sugar concentration in order to discover the optimum conditions for the biomass production from date extract. According to the experimental results, a maximum biomass concentration of 40 g/L was obtained at the optimum condition of temperature (32.9 °C), pH (5.35) and total reducing sugars (70.93 g/L).

In addition, among three unstructured kinetic models, both Monod and Verhulst models represent the experimental data of biomass production kinetics; nevertheless, the Verhulst model was the most suitable model to signify the baker’s yeast production on date extract medium.

## Figures and Tables

**Table 1:** Coded levels and real values of studied variables.

**Variables**	**Coded Levels**				
	−α	−1	0	+1	+α
	**Real Values**				
*X*_1_ = Temperature (°C)	27	29	33	37	39
*X*_2_ = Initial pH	2.4	3.6	5.5	7.3	8.6
*X*_3_ = concentration of sugars (g/L)	1	44.1	107.5	170.9	214

In the central composite design, the −1 and +1 correspond to the lower and the higher level, respectively. The value 0 represents the central value of the rangeand α has the value of 1.68 (α = ∜*N_f_*, where *N_f_* is a number of experiments).

**Table 2:** The central composite experimental design (CCD) matrix for different variables (coded levels).

Experiments	Coded Levels		
	X_1_	X_2_	X_3_
01	−1	−1	−1
02	+1	−1	−1
03	−1	+1	−1
04	+1	+1	−1
05	−1	−1	+1
06	+1	−1	+1
07	−1	+1	+1
08	+1	+1	+1
09	−1.68	0	0
10	+1.68	0	0
11	0	−1.68	0
12	0	+1.68	0
13	0	0	−1.68
14	0	0	+1.68
15	0	0	0
16	0	0	0
17	0	0	0
18	0	0	0
19	0	0	0
20	0	0	0

The CCD matrix is composed of a complete factorial design, 2^3^; two axial points on the axis of each design variable at a distance of α = 1.682 from the design center and 5 points at the domain center. The actual experimental values corresponding to the coded levels used for the creation of the experiment matrix are presented below (Table 3).

**Table 3:** Actual values for the three independent variables.

Experiments	Actual Values		
	Temperature (°C)	Initial pH	Sugars Concentration (g/L)
01	29	3.6	44.1
02	37	3.6	44.1
03	29	7.3	44.1
04	37	7.3	44.1
05	29	3.6	170.9
06	37	3.6	170.9
07	29	7.3	170.9
08	37	7.3	170.9
09	27	5.5	107.5
10	39	5.5	107.5
11	33	2.4	107.5
12	33	8.6	107.5
13	33	5.5	1
14	33	5.5	214
15	33	5.5	107.5
16	33	5.5	107.5
17	33	5.5	107.5
18	33	5.5	107.5
19	33	5.5	107.5
20	33	5.5	107.5

**Table 4:** Unstructured kinetic models to determinate the kinetic parameters.

Kinetic Models	Equations	Linearized Form	Description	Symbols
Monod	$\mu =\mu_{max}\frac{S}{S+K_{S}}$	$\frac{1}{\mu }\;=\frac{K_{S}}{\mu_{max}}\frac{1}{S}+\frac{1}{\mu_{max}}$	Monod kinetic model is a substrate concentration dependent.	μ: is the specific growth rate (h^−1^). $\mu_{max}$: is the maximum specific growth rate (h^−1^). $K_{S}$: is the half-saturation constant (g/L). *S*: is the concentration in limiting substrate (g/L). X: is the biomass concentration (g/L). $X_{m}$: is the Maximum biomass concentration (g/L).
Verhulst	$\mu =\mu_{max}\left(1-\frac{X}{X_{m}}\right)$	$\mu =\mu_{max}-\frac{\mu_{max}}{X_{m}}X$	Verhulst kinetic model is an unstructured model depends on biomass concentration.	
Tessier	$\mu =\mu_{max}\left(1-e^{-KsS}\right)$	$\ln\mu =\frac{1}{K_{s}}S+\ln\mu_{max}$	Tessier is an unstructured model for a substrate concentration dependent.	

**Table 5:** The central composite design for biomass production.

Experiments	Coded Levels			Real Values			(*Y_i_*): Biomass (g/L)	
	*X_1_*	*X_2_*	*X_3_*	Temperature (°C)	Initial pH	Concentration of Sugar (g/L)	Observed Mean Values *	Predicted Values
01	−1	−1	−1	29	3.6	44.1	24.07	23.99
02	+1	−1	−1	37	3.6	44.1	15.99	17.45
03	−1	+1	−1	29	7.3	44.1	25.70	27.80
04	+1	+1	−1	37	7.3	44.1	15.79	20.98
05	−1	−1	+1	29	3.6	170.9	28.40	25.05
06	+1	−1	+1	37	3.6	170.9	29.86	29.59
07	−1	+1	+1	29	7.3	170.9	20.78	21.16
08	+1	+1	+1	37	7.3	170.9	23.51	25.42
09	−1.68	0	0	27	5.5	107.5	22.61	24.06
10	+1.68	0	0	39	5.5	107.5	26.20	22.15
11	0	−1.68	0	33	2.4	107.5	26.00	28.21
12	0	+1.68	0	33	8.6	107.5	32.72	27.90
13	0	0	−1.68	33	5.5	1	25.37	21.09
14	0	0	+1.68	33	5.5	214	24.04	25.71
15	0	0	0	33	5.5	107.5	40.00	40.07
16	0	0	0	33	5.5	107.5	40.00	40.07
17	0	0	0	33	5.5	107.5	40.00	40.07
18	0	0	0	33	5.5	107.5	40.00	40.07
19	0	0	0	33	5.5	107.5	40.00	40.07
20	0	0	0	33	5.5	107.5	40.00	40.07

* Each experiment was carried out twice and the average value is used here.

**Table 6:** Estimated regression coefficients of t and *p*-values of the model.

Terms	Coefficients	Square Error	*t*-Value	*p*
β_0_	40.0744	1.3912	28.806	0.000
β_1_	−0.5684	0.9230	−0.616	0.552
β_2_	−0.0907	0.9230	−0.098	0.924
β_3_	1.3739	0.9230	1.488	0.167
**β_11_**	**−6.0000**	**0.8985**	**−6.677**	**0.000**
**β_22_**	**−4.2481**	**0.8985**	**−4.728**	**0.001**
**β_33_**	**−5.8939**	**0.8985**	**−6.559**	**0.000**
β_12_	−0.0700	1.2060	−0.058	0.955
**β_13_**	**2.7725**	**1.2060**	**2.299**	**0.044**
β_23_	−1.9250	1.2060	−1.596	0.142

*R*^2^ = 91.1%, *R*^2^ (adj) = 83.16%, S = 3.41104, PRESS = 884.951.

**Table 7:** Analysis of variance (ANOVA).

Source	DF	Seq SS	Adj SS	Adj MS	*F*	*p*
Regression	9	1196.65	1196.65	132.961	**11.43**	**0.000**
Linear	3	30.30	30.30	10.101	0.87	0.489
A	1	4.41	4.41	4.412	0.38	0.552
B	1	0.11	0.11	0.112	0.01	0.924
C	1	25.78	25.78	25.779	2.22	0.167
Square	3	1075.17	1075.17	358.390	30.80	0.000
A*A	1	379.27	518.80	518.799	44.59	0.000
B*B	1	195.28	260.07	260.071	22.35	0.001
C*C	1	500.62	500.62	500.618	43.03	0.000
Interaction	3	91.18	91.18	30.393	2.61	0.109
A*B	1	0.04	0.04	0.039	0.00	0.955
A*C	1	61.49	61.49	61.494	5.29	0.044
B*C	1	29.64	29.64	29.645	2.55	0.142
Residual Error	10	116.35	116.35	11.635		

DF: degrees of freedom; Seq SS: sequential sum of squares; Adj SS: adjusted, sum of squares; AdjMS: adjusted, mean of squares F: Fischer’s variance ratio; P: probability value.

**Table 8:** Kinetic parameters of *S. cerevisiae* growth and substrate utilization using unstructured models.

Kinetic Models		Parameters Estimation		
	*R^2^*	*Ks* (g/L)	*μ_max_* (h^−1^)	*X_m_*
Monod	0.945	0.228	0.496	-
Verhulst	0.981	-	0.376	15.04
Tessier	0.979	−9.434	0.408	
